# Microbiota-miR-101 interactions in obesity-associated colorectal cancer: from barrier dysfunction to precision therapeutic strategies

**DOI:** 10.3389/fphar.2026.1850919

**Published:** 2026-06-12

**Authors:** Sidharth Prasad Mishra, Richard Jacobson, Bo Wang, Santosh Prajapati, Paul Sanberg, Christian Brechot, Shalini Jain, Hariom Yadav

**Affiliations:** 1 USF Center for Microbiome Research, Microbiomes Institute, University of South Florida, Tampa, FL, United States; 2 Department of Neurosurgery, Brain and Spine, University of South Florida, Tampa, FL, United States; 3 Center for Excellence of Aging and Brain Repair, University of South Florida, Tampa, FL, United States; 4 Byrd Alzheimer’s Institute, University of South Florida Morsani College of Medicine, Tampa, FL, United States; 5 Tampa General Hospital Cancer Institute, University of South Florida, Tampa, FL, United States; 6 Department of Surgery, University of South Florida, Tampa, FL, United States; 7 Department of Biomedical and Chemical Engineering and Sciences, Florida Institute of Technology, Melbourne, FL, United States

**Keywords:** chronic inflammation, colorectal cancer, ethanolamine, leaky gut, microRNA, obesity, T2D, miR-101 family

## Abstract

Colorectal cancer (CRC) remains a leading cause of cancer-related morbidity and mortality worldwide, with obesity recognized as a major modifiable risk factor. Obesity-associated CRC is characterized by systemic low-grade inflammation, altered lipid metabolism, and gut microbial dysbiosis, all of which converge to create a pro-inflammatory niche. Emerging evidence implicates murine miR-101a/b, an ortholog of the human miR-101 family, as a key molecular mediator linking metabolic dysfunction, promoting inflammation, endotoxemia, and affecting epithelial homeostasis. Traditionally, the miR-101 family is considered a tumor suppressor by repressing oncogenes such as EZH2, MCL-1, and COX-2; miR-101a appears to exhibit a paradoxical microenvironment-modulating role in obese colon. Recent studies demonstrate that elevated dietary and microbiota-derived ethanolamine induces miR-101a overexpression in colonic epithelial cells. Mechanistically, miR-101a directly destabilizes the mRNA encoding the tight junction protein (ZO-1; TJP1), thereby impairing epithelial barrier integrity, increasing intestinal permeability, and promoting chronic inflammation. The chronic inflammation promotes epithelial proliferation, generates mutagenic reactive oxygen species, and activates pro-survival pathways such as STAT3 and AKT, collectively contributing to a tumor-permissive microenvironment that may support adenoma initiation and progression. The resulting chronic inflammatory milieu promotes epithelial stress, proliferative signaling, and accumulation of DNA damage, contributing to conditions that favor colorectal carcinogenesis. Importantly, this ethanolamine-miR-101a axis represents a novel mechanistic link between diet, microbiota, and cancer biology. Translationally, miR-101a holds promise as a biomarker of early barrier dysfunction and CRC risk, as detectable in tissue, serum, or fecal samples. Furthermore, microbiome-targeted interventions, dietary modifications, or direct inhibition of miR-101a may offer innovative therapeutic strategies. Collectively, these findings support the development of precision microbiome-miRNA-based approaches and highlight the importance of context-dependent miRNA regulation in obesity-associated CRC.

## Graphical Abstract

**Figure:**
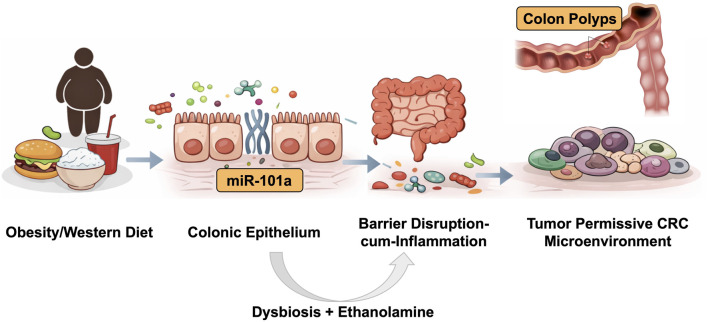

## Introduction

Colorectal cancer (CRC) remains one of the leading causes of cancer-related mortality worldwide, accounting for more than 1.9 million new cases and over 900,000 deaths annually (Akimoto et al., 2021). Although widespread screening and advances in systemic therapies have improved survival, the incidence of early-onset CRC (diagnosed before 50 years of age) has risen steadily over the past 2 decades, particularly in Westernized populations (Akimoto et al., 2021). Obesity is now recognized as a major independent risk factor for CRC, with umbrella meta-analyses reporting approximately 18%–32% increased CRC risk in overweight individuals and up to 30%–50% higher risk in obese populations compared with normal-weight individuals (Mandic et al., 2023). Emerging evidence suggests that obesity-associated CRC is not solely a mutation-driven disease, but also a microbiota-influenced inflammatory disorder characterized by chronic low-grade inflammation, epithelial barrier dysfunction, altered microbial ecology, and dysregulated host-microbiota signaling (Arima et al., 2022; Lednovich et al., 2024; Lednovich et al., 2022). Western-style diets rich in fat and red meat further promote gut microbial dysbiosis and expansion of pro-carcinogenic microbial communities, thereby contributing to conditions associated with colorectal carcinogenesis (Arima et al., 2022). Despite increasing recognition of obesity-associated CRC as a microbiota-influenced disease, therapeutic strategies specifically targeting microbiota-driven epithelial barrier dysfunction and inflammatory signaling remain limited. This review outlines a mechanistic framework linking diet-associated alterations in the gut microbiota, microbial metabolite signaling, and miRNA-mediated regulation within the carcinogenesis-supportive environment of obesity-associated CRC.

MicroRNAs (miRNAs) are small non-coding RNAs that regulate post-transcriptional gene expression and play critical roles in cellular proliferation, apoptosis, immune regulation, and stress adaptation (He and Hannon, 2004; Li et al., 2025; Xiao and Rajewsky, 2009; Chaiwangyen et al., 2025). Dysregulated miRNA signaling is increasingly recognized as an important contributor to inflammation-associated carcinogenesis, with certain miRNAs exhibiting context-dependent functions depending on the metabolic and inflammatory microenvironment (Singh et al., 2025; Otmani et al., 2024; Sareen et al., 2025). Among these, the miR-101 family is particularly relevant to obesity-associated CRC because of its role at the intersection of epithelial barrier regulation, microbiota-responsive metabolism, inflammatory signaling, and epigenetic remodeling. In mice, the miR-101 family includes miR-101a and miR-101b, orthologs of the human miR-101-1 and miR-101-2 families (Chandramouli et al., 2012; Cohen et al., 2017; Dudakovic et al., 2022; Liu et al., 2014; de Sande et al., 2023; Morita et al., 2014). Historically, members of the miR-101 family have been characterized primarily as tumor suppressors by regulating pathways involved in epigenetic remodeling, apoptosis, inflammatory signaling, and cellular proliferation, including Enhancer of Zeste Homolog 2 (EZH2), Cyclooxygenase-2/Prostaglandin-Endoperoxide Synthase 2 (COX-2/PTGS2), DNA Methyltransferase 3A and DNA Methyltransferase 3B (DNMT3A/B), and Myeloid Cell Leukemia Sequence 1 (MCL-1) (Cohen et al., 2017; Wang et al., 2016a; Vella et al., 2015; Xu et al., 2015; Cao et al., 2011; Cao et al., 2010; Smits et al., 2011; Hillyar et al., 2022; Gómez-Acebo et al., 2025; Huang et al., 2025; Liu et al., 2022; Pastena et al., 2024). However, accumulating evidence suggests that the miR-101 family exhibits context-dependent functions under conditions of metabolic dysregulation, chronic inflammation, and epithelial barrier dysfunction, particularly in obesity-associated CRC (Tessitore et al., 2014; Schnekenburger and Diederich, 2012; Tâlvan et al., 2024; Mishra et al., 2023; Tarek et al., 2025).

Obesity and/or type-2 diabetes (T2D) are associated with systemic inflammation, insulin resistance, altered lipid metabolism, and dysbiosis of the gut microbiota, all of which contribute to colorectal carcinogenesis (Wu and Ballantyne, 2020; Jia et al., 2021; Semo et al., 2024; Yarahmadi et al., 2024; Upadhyay et al., 2024; Nagpal et al., 2020; Glaros et al., 2025; Dixon et al., 2023). The CRC microenvironment is enriched with pro-inflammatory lipid mediators and deficient in pro-resolving mediators, leading to persistent, non-resolving inflammation (Upadhyay et al., 2024; Soundararajan et al., 2025). In this condition, miR-1011/2 expression may increase rather than decrease, leading to downstream effects that deviate from its classical tumor-suppressive role. Diets enriched in fat and red meat increase luminal ethanolamine concentrations, a membrane phospholipid-derived metabolite that serves as a carbon and nitrogen source for multiple gut bacteria (Zhou et al., 2017; Bourdeau-Julien et al., 2023). Elevated intestinal ethanolamine has been shown to induce miR-101a expression in colonic epithelial cells (Mishra et al., 2023), supporting a mechanistic link between diet, microbial metabolism, and host transcriptomic regulation. Consistent with its clinical relevance, increased expression of the miR-101 family has been associated with CRC severity, invasiveness, and anatomical localization (Tâlvan et al., 2025). Additional studies further demonstrate that dysregulated miR-101 family signaling contributes to epithelial permeability, inflammatory activation, migration, and tumor progression across multiple cancer models (Liu et al., 2014; Tâlvan et al., 2025; Shukla et al., 2024). Mechanistically, elevated miR-1011/2 expression can destabilize tight-junction-associated proteins, including vascular endothelial (VE)-cadherin and claudin-5, thereby promoting tissue permeability and inflammatory signaling (Shukla et al., 2024). Importantly, our recent findings demonstrated that microbiota-derived ethanolamine induces miR-101a overexpression in intestinal epithelial cells, leading to destabilization of zonula occludens-1 (ZO-1) mRNA, increased gut permeability, microbial translocation, and chronic inflammatory activation in obese and diabetic conditions (Mishra et al., 2023). Despite advances in screening and systemic therapies, clinically effective strategies specifically targeting microbiota-driven epithelial barrier dysfunction and inflammatory signaling in obesity-associated CRC remain limited.

Emerging evidence further supports the relevance of ethanolamine metabolism in cancer biology. In silico analyses demonstrated that ethanolamine kinase (ETNK)1, a key enzyme in phosphatidylethanolamine biosynthesis, is overexpressed in hepatocellular carcinoma and associated with poor clinical prognosis (Rahimi-Farsi et al., 2025). Although derived from liver cancer studies, these findings support the broader concept that dysregulated ethanolamine metabolism may influence epithelial signaling and tumor progression in other metabolically dysregulated diseases, including obesity-associated CRC. However, whether luminal or tissue ethanolamine concentrations directly correlate with miR-101 family expression, inflammatory biomarkers, or disease progression in human obesity-associated CRC remains unclear. This represents an important translational gap and highlights the need for integrated metabolomic, microbiome, and transcriptomic studies in obesity-associated CRC patient cohorts. Collectively, these findings position the ethanolamine-miR-101 axis as a microbiota-responsive regulatory pathway integrating diet, dysbiosis, epithelial barrier dysfunction, transcriptomic regulation, and chronic inflammatory signaling in obesity-associated CRC. Understanding this metabolically dependent signaling network may facilitate the development of precision microbiome-targeted and RNA-directed therapeutic strategies for obesity-associated colorectal carcinogenesis.

## Genomic organization and sequence features of the miR-101 family

In mammals, the miR-101 family is encoded by two conserved loci that produce the same predominant mature effector strands, mature miR-101 family-derived 3p and 5p strands (5′-UAC​AGU​ACU​GUG​AUA​ACU​GAA-3′) (microRNA 101a, 2025a). In humans, the precursors map to miR-101–1 on chr1p31.3 and miR-101–2 on chr9p24.1; in mice, the orthologues loci correspond to miR-101a on chr4 (negative strand; GRCm39: ∼101,204,142–101,204,224) and miR-101b on chr19 (positive strand; ∼29,112,679–29,112,775) (Perez et al., 2024; Cao et al., 2024; microRNA 101a, 2025b; microRNA 101a, 2026a; microRNA 101a, 2025b). Within the human genome, miR-101 derives from two primary precursors, miR-101–1 (75 bp) and miR-101–2 (79 bp), both of which are essential for its biogenesis. In mice, miR-101b is embedded within the intron of RNA Terminal Phosphate Cyclase-Like (Rcl)1, whereas miR-101a is intergenic, an arrangement that directly contributes to subtle differences in transcriptional control and co-regulation with host-gene programs (Zeng et al., 2023). Biogenesis follows the canonical microRNA processing pathway: RNA polymerase II transcription, nuclear cropping by Drosha-DiGeorge Critical Region (DGCR8), cytoplasmic cleavage by Dicer, and Argonaute loading into the RNA-Induced Silencing Complex (RISC) (Cao et al., 2025; SiamiGorji et al., 2020). Although both arms are detectable, most tissues preferentially load the 3p arm, establishing miR-101–3p as the principal functional strand for target repression. Sequence variation introduces additional regulatory complexity. Single-nucleotide variants within the hairpin (e.g., basal junction, apical loop, dicer processing sites) can shift microprocessor/dicer efficiency, thereby affecting mature miRNA abundance and 5p/3p arm selection (Chen et al., 2014; Omariba et al., 2020; Miao et al., 2016). Variants within the seed regions (nts two to eight of miR-101-3p) are especially consequential, as they redefine the targetome, simultaneously extinguishing canonical sites (e.g., in Enhancer of Zeste Homolog [EZH]2 or Post-Transcriptional Gene Silencing [PTGS]2/Cyclooxygenase [COX]-2) and creating novel interactions in unrelated transcripts (Cao et al., 2010; Su et al., 2009). Population resources catalog multiple variants in and around miR-101-1/2; some of which have been associated with altered cancer risk, underscoring their potential functional impact (Cao et al., 2025). Complementing miRNA-centric variation, 3′-UTR polymorphisms (“miR-eQTLs”) within target genes can gain or lose miR-101 recognition motifs, which are critically responsible for repression across individuals, tissues, and developmental stages.

Expression atlases reveal a broad abundance of miR-101 family-derived nature strands. In the mouse brain, miR-101a/b levels rise from late embryogenesis (∼E16) through early postnatal stages (∼P12), consistent with roles in neuronal maturation and circuit refinement (Lippi et al., 2016). In adults, miR-101-1/2 is readily detected across epithelial, stromal, and immune compartments, with absolute levels tuned by hormonal, inflammatory, and metabolic cues (Lippi et al., 2016; Kavakiotis et al., 2022; Panwar et al., 2017). Functionally, miR-101 family derived mature miRNAs identified to be a critically important in regulating the chromatin state, eicosanoid/inflammatory tone, cell survival, and cytoskeletal dynamics-with repeatedly validated targets including EZH2, MCL-1, PTGS2/COX-2, and disease-stage-specific effectors such as FBJ Murine Osteosarcoma Viral Oncogene (FOS), Stathmin (STMN)1, DNMT3A, Ras-related C3 botulinum toxin substrate (RAC)1, SRY (Sex-determining Region Y)-Box (SOX)9, and Cyclin-Dependent Kinase (CDK)8 (Cao et al., 2010; Su et al., 2009; Tanaka et al., 2009; Konno et al., 2014; Wang C. Z. et al., 2018; Ochs et al., 2011; Li et al., 2015). These interactions typically impose anti-proliferative and anti-inflammatory constraints in epithelial tissues. However, tissue state (e.g., obesity-associated inflammation, cytokine milieu, metabolite availability) can invert net outcomes by reshaping competing RNA networks and transcriptional baselines. Aberrant expression of the miR-101 family profoundly influences genomic stability by targeting multiple classes of genes involved in DNA repair, chromatin regulation, inflammation, and barrier integrity (Cao et al., 2010; Mishra et al., 2023; Mishra and Singh, 2013; Shukla et al., 2024; Friedman et al., 2009). At the level of the DNA damage response (DDR), miR-101-1/2 directly represses ataxia-telangiectasia mutated (ATM) and Protein Kinase, DNA-Activated, Catalytic Subunit (PRKDC [DNA-PKcs]), impairing double-strand break repair and thereby sensitizing epithelial cells to DNA damage and mutational accumulation (Camfield et al., 2024; Yan et al., 2010). In parallel, miR-101 family members regulate chromatin modifiers, including EZH2, Disruptor of Telomeric Silencing 1-Like (DOT1L), and DNMT3A/3B, leading to altered histone methylation and DNA methylation landscapes that promote epigenetic instability and oncogenic transcriptional reprogramming (Liu et al., 2022; Huang et al., 2021; Man et al., 2022; Ma et al., 2023; Szczepanek and Tretyn, 2023; Varambally et al., 2008). Cytoskeletal fidelity is also influenced through repression of STMN1, which disrupts microtubule dynamics and predisposes cells to aneuploidy (Zhu et al., 2018; Xu et al., 2013; Sun et al., 2015). Collectively, these findings position miR-101a as a context-dependent regulator of epithelial stress responses, inflammatory signaling, and tumor-permissive microenvironmental remodeling that does not directly induce point mutations but instead establishes a mutagenic microenvironment by suppressing DNA repair, promoting epigenetic deregulation, driving prostaglandin-mediated inflammation, and disrupting barrier function, ultimately contributing to conditions that may favor colorectal tumor initiation and progression.

### Induction of miR-101a by metabolic and gut barrier dysregulation

The microenvironment-specific pro-inflammatory effects of miR-101a are closely linked to diet and metabolites generated by the gut microbiota (Mishra et al., 2023). In obesity-associated CRC, these molecular effects converge with barrier dysfunction, where miR-101a-driven barrier dysfunction, primarily through tight-junction disruption (e.g., ZO-1), may be accompanied by secondary alterations in mucus-layer organization, although direct regulation of goblet cell differentiation or MUC2 expression by miR-101a has not been established. This barrier dysfunction permits microbial translocation and activation of Nuclear Factor kappa-light-chain-enhancer of activated B cells (NF-κB)/Interleukin (IL)-6/(Signal Transducer and Activator of Transcription) STAT3 signaling (Tonetti et al., 2024; Stanforth et al., 2024; Schulz-Heddergott et al., 2018; Yu et al., 2014). This inflammatory loop sustains the production of reactive oxygen and nitrogen species (ROS/RNS), amplifying DNA damage and accelerating the clonal expansion of mutated epithelial cells. Recent investigations demonstrate that intestinal ethanolamine, a phospholipid precursor derived from dietary phosphatidylethanolamine and abundant in high-fat and high-meat diets (Zhou et al., 2017; Patel and Witt, 2017; Sogame et al., 2025; Zhou et al., 2018; Everard et al., 2019; Elmihi et al., 2025; Shi et al., 2025). Ethanolamine is a preferred nutrient source for several gut bacterial taxa, including opportunistic pathogens and dysbiosis-associated organisms that are enriched during dysbiosis (Barnes et al., 2024; Rowley et al., 2018; Thiennimitr et al., 2011; Ormsby et al., 2019; Pacheco and Sperandio, 2015; Lengfelder et al., 2019; Garsin, 2010; Anderson et al., 2015). In conditions of obesity and metabolic syndrome, dysbiotic microbiota increases the availability of ethanolamine in the colonic lumen (Mishra et al., 2023; Thiennimitr et al., 2011; Fang et al., 2025; Breton et al., 2022; Zeng et al., 2017; Mishra et al., 2021). This metabolite acts as more than a passive nutrient and functions as a signaling molecule linking diet to host transcriptional regulation (Mishra et al., 2023; Mishra S. et al., 2025). Elevated ethanolamine stimulates upregulation of miR-101a in colonic epithelial cells, thereby altering the delicate balance between epithelial homeostasis and injury responses. Unlike the canonical tumor-suppressive functions of miR-101a observed in non-obese conditions, chronic upregulation in obesity skews epithelial biology toward barrier dysfunction and inflammatory remodeling (Mishra et al., 2023). This metabolic induction of miR-101a positions it as a molecular bridge connecting nutrition, dysbiosis, and tumor-permissive epithelial and inflammatory changes.

One of the earliest consequences of ethanolamine-induced miR-101a upregulation is disruption of epithelial barrier integrity. In obese and diabetic mouse models, ethanolamine-induced miR-101a expression significantly reduced ZO-1 expression and compromised barrier integrity, thereby enhancing microbial translocation and inflammation (Mishra et al., 2023). Tight-junction disruption represents a well-established mechanism underlying “leaky gut” during obesity-associated metabolic dysfunction and inflammatory disease (Genua et al., 2021; Neurath et al., 2025; Kearns, 2025; Lei et al., 2024; He et al., 2025; Matar et al., 2024; Dmytriv et al., 2024). In addition to tight-junction alterations, barrier integrity may also be influenced by changes in mucus-layer organization and goblet cell biology. The intestinal mucus barrier is primarily composed of mucin (MUC)2, which forms the structured inner and outer mucus layers that spatially segregate luminal microbes from epithelial surfaces (Johansson et al., 2011). Although global disruption of miRNA biogenesis has been shown to alter intestinal epithelial differentiation and goblet cell function (McKenna et al., 2010), direct evidence linking miR-101a specifically to MUC2 suppression or goblet-cell differentiation remains limited. Therefore, mucus-layer alterations should currently be viewed as a potential complementary mechanism rather than the primary evidence-supported pathway of miR-101a-mediated barrier dysfunction.

**FIGURE 1:**
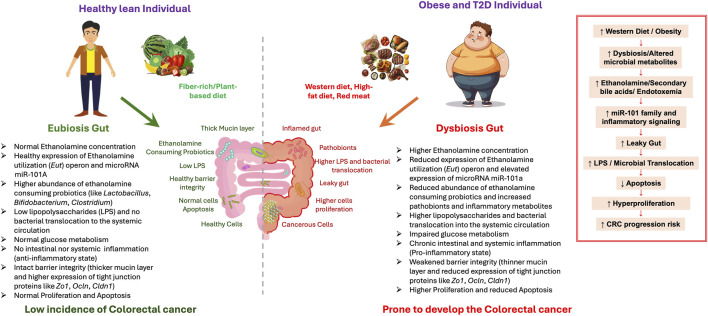
Schematic representation contrasting gut ecosystem states in a healthy lean individual (left) versus an obese, type 2 diabetic (T2D) individual (right). In lean subjects, a eubiotic gut microbiota maintains balanced ethanolamine concentrations and supports efficient ethanolamine utilization pathways (Eut operon), thereby preventing overexpression of the context-dependent tumor-promoting effects of miR-101a. Intact mucus barrier integrity, abundant commensal taxa (e.g., *Lactobacillus*, *Bifidobacterium*, *Clostridium*), and regulated inflammatory responses collectively preserve epithelial homeostasis. This microenvironment is characterized by reduced pro-inflammatory signaling, proper metabolic regulation, and protection against DNA damage, preventing colorectal cancer initiation. By contrast, in obese and T2D individuals, dysbiosis results in elevated luminal ethanolamine and impaired utilization, leading to pathological miR-101a upregulation. Primary disruption of epithelial tight-junction integrity (ZO-1), with potential secondary effects on mucus-layer organization that weakens the mucus barrier integrity, allowing microbial translocation and sustained activation of inflammatory pathways. Chronic cytokine signaling fosters epithelial hyperproliferation, genomic instability, and impaired apoptosis, while simultaneous angiogenic and immunosuppressive cues create a tumor-permissive niche. These changes predispose obese and metabolically dysregulated hosts to conditions associated with increased risk of colorectal adenomas and carcinoma.

Alterations in mucus-layer organization may occur secondary to epithelial barrier disruption and inflammatory stress, further increasing microbial-epithelial interactions and dysbiosis (Okumura and Takeda, 2024; Song et al., 2023; Zhou et al., 2025; Mishra et al., 2024; Bergstrom et al., 2010; Johansson et al., 2014; van der Post et al., 2019; Jiang et al., 2025; Guagliano et al., 2025). However, direct evidence linking miR-101a to reduced MUC2 expression or goblet-cell dysfunction remains limited. Therefore, mucus-layer alterations should be interpreted as a secondary or complementary consequence rather than a primary mechanism of miR-101a activity. However, this disruption of the epithelial and mucosal barrier represents a biologically plausible early event associated with CRC-relevant pathogenesis (Genua et al., 2021; Neurath et al., 2025; Kearns, 2025). Barrier impairment creates a permissive environment for microbial translocation, allowing endotoxins, such as lipopolysaccharide (LPS), to cross into the lamina propria and the systemic circulation (Neurath et al., 2025; Lei et al., 2024; He et al., 2025; Matar et al., 2024; Dmytriv et al., 2024). The resulting endotoxemia perpetuates immune activation and contributes to systemic low-grade inflammation, a hallmark of obesity-associated diseases (Mazaheri-Tehrani et al., 2025; Starbæk et al., 2025; Hu J. et al., 2024; Dimeji and Ayodeji, 2025; Jacobson et al., 2025). Figure 1 illustrates the proposed mechanistic framework in which obesity-associated dysbiosis, and ethanolamine metabolism induce miR-101a expression, leading to ZO-1 destabilization, epithelial barrier dysfunction, microbial translocation, activation of inflammatory signaling, and conditions favoring colorectal carcinogenesis. Moreover, the loss of mucin alters the spatial distribution of the microbiota, promoting closer interactions between microbes and the epithelium, which further exacerbate dysbiosis (Bourdeau-Julien et al., 2023; Mishra et al., 2024; Dmytriv et al., 2024; Fang et al., 2021; Rath and Haller, 2022; Mishra P. et al., 2025). Thus, miR-101a-mediated suppression of ZO-1 mRNA stability dismantles barrier function, a key protective mechanism of the gut, transforming the mucosal interface from a defensive barrier into a zone of persistent microbial challenge.

In addition to ethanolamine, several microbiota-derived metabolites influence epithelial homeostasis, inflammatory signaling, and tumor microenvironment remodeling in CRC. Short-chain fatty acids (SCFAs), particularly butyrate, generally support epithelial barrier integrity and anti-inflammatory signaling through histone deacetylase inhibition and G-protein-coupled receptor activation, whereas dysregulated secondary bile acids, such as deoxycholic acid, promote oxidative stress, DNA damage, Wnt/β-catenin activation, and pro-inflammatory signaling. Microbial metabolites derived from tryptophan, polyamines, and lipid metabolism further modulate epithelial proliferation, immune responses, and carcinogenesis-associated signaling pathways. However, direct mechanistic evidence linking these metabolites to miR-101 family regulation remains limited. Currently, ethanolamine is the most strongly supported microbiota-derived metabolite directly associated with miR-101a induction and epithelial barrier dysfunction in obesity-associated dysbiotic conditions.

### Chronic inflammation as a contributor of malignant transformation via miR-101a

The epithelial barrier defects induced by miR-101a create fertile ground for sustained inflammation, which in turn may contribute to processes associated with tumor initiation and progression. Microbial translocation engages pattern recognition receptors (PRRs), such as Toll-like receptors (TLRs), on epithelial and immune cells, triggering intestinal and systemic inflammation (Mukherjee et al., 2024; Chen et al., 2024; Clarke et al., 2011). This leads to robust transcription of pro-inflammatory cytokines, including Tumor Necrosis Factor (TNF)-α, IL-6, and IL-1β, which amplify local and systemic immune responses (Garavaglia et al., 2024; Robles-Vera et al., 2025; Lei et al., 2025). In fact, members of the miR-101 family have been shown to directly target the 3′-UTR of PTGS2/COX-2, thereby repressing COX-2 translation and reducing prostaglandin synthesis in several cellular circumstances (Desind et al., 2023; Hao et al., 2011). Although miR-101-1/2 itself represses COX-2 expression under many conditions, in obesity-associated chronic inflammation, it activates transcription factors such as NF-κB, STAT3, and AP-1, which directly regulate the transcription of inflammatory genes, including COX-2 (PTGS2). Sustained activation of these pathways by cytokines, adipokines, and metabolic stress promotes COX-2 expression and prostaglandin production, thereby amplifying inflammatory signaling and contributing to colorectal and pancreatic tumorigenesis (Zhang et al., 2020; Liu et al., 2017; Gong et al., 2014). Moreover, elevated COX-2 activity promotes angiogenesis, inhibits apoptosis, and fosters immune evasion, thereby contributing to a pro-neoplastic environment (Che et al., 2021; Liu et al., 2022). Furthermore, chronic inflammation stimulates the production of ROS/RNS by infiltrating immune cells (Chandimali et al., 2025; Morris et al., 2022; Manoharan et al., 2024). These reactive ROS/RNS molecules induce DNA damage and mutagenic stress in epithelial cells, thereby potentially increasing mutagenic stress and accumulation of oncogenic alterations (Li et al., 2024; Shahgoli et al., 2024; Farmanbar et al., 2023; Thatikonda et al., 2023; Grzelakowska et al., 2024; Seiwert et al., 2020; Irrazabal et al., 2020). In addition to inflammatory signaling, several intestinal pathobionts produce microbial genotoxins that directly contribute to colorectal carcinogenesis. Colibactin-producing *Escherichia coli*, enterotoxigenic *Bacteroides fragilis*, and cytolethal distending toxin-producing bacteria induce DNA damage, genomic instability, epithelial proliferation, and chronic inflammatory activation. These microbial factors synergize with obesity-associated barrier dysfunction and endotoxemia to establish a tumor-permissive microenvironment. Although the direct interactions between microbial genotoxins and miR-101 family regulation remain incompletely defined, these pathways likely converge through shared inflammatory and epithelial-stress signaling networks involving NF-κB, STAT3, and oxidative stress responses.

This chronic inflammatory state not only sustains epithelial injury but also promotes cycles of injury and regenerative proliferation, further increasing the probability of malignant transformation (Xie et al., 2025; Wen et al., 2022; Tripathi et al., 2025; Greten and Grivennikov, 2019). Local immune cells experience exhaustion, and populations of anti-tumor cytotoxic T-cells decline (Chi et al., 2023). Importantly, inflammation-induced activation of STAT3 and related oncogenic pathways promotes survival signaling and resistance to apoptosis in epithelial cells (Voshagh et al., 2024; Hu Y. et al., 2024; Rahbar Farzam et al., 2024), consolidating the metabolism-dependent inflammatory role of miR-101a in the inflamed gut. This inflammatory microenvironment promotes cycles of epithelial injury and regenerative proliferation. The colon is a tissue characterized by rapid turnover, with epithelial cells replenished by stem cells located at the base of the crypts. In the presence of persistent barrier stress and inflammation, stem cells undergo hyperproliferation to repair damaged mucosa (Choi and Augenlicht, 2024; Liu et al., 2025). This process, while initially protective, increases the probability of replication errors, clonal expansion, and selection of mutant populations. Within this hyperproliferative condition, canonical oncogenic pathways such as STAT3, NF-κB, COX-2/Prostaglandin E_2_ (PGE2), and Wingless Integration-1 (Wnt)/β-catenin are activated, synergizing with accumulating genetic mutations to drive the transition from normal epithelium to dysplastic adenomas (Shahgoli et al., 2024; Good et al., 2025; Cineus et al., 2025; Morrison et al., 2024). Importantly, miR-101a acts as an upstream contributor to this vicious cycle by compromising epithelial barrier integrity (ZO-1), with potential secondary effects on mucus-layer organization, and by facilitating microbial translocation, thereby perpetuating the continuous activation of inflammatory and proliferative signaling pathways (Mishra et al., 2023; Li et al., 2024; Song et al., 2024). As adenomas develop, miR-101a continues to influence tumor progression by remodeling the tumor microenvironment. The validated gene and protein targets of miR-101a, functionally implicated in diverse disease conditions, are listed in Table 1. Persistent COX-2 activity and cytokine signaling stimulate neovascularization, ensuring adequate oxygen and nutrient supply for expanding lesions (Deng et al., 2021; Geindreau et al., 2022; Jacobson et al., 2020). Inflammatory mediators and pathogenic bacteria remodel the extracellular matrix, weakening cell adhesion and facilitating epithelial invasion into deeper tissue layers.

**TABLE 1:** Validated gene and protein targets of mIR-101a and their functional roles in cancer and disease contexts.

Gene/Protein	Function	Ref.
E-cadherin	Overexpressed miR-101 suppresses DNMT3A, thereby restoring E-cadherin and inhibiting proliferation and migration in MDA-MB-231 breast cancer cells	Liu et al. (2016a)
EZH2	Overexpression of miR-101a-3p in the amygdala increases anxiety-like behavior in rats by repressing Ezh2	Cohen et al. (2017)
	miR-101 promotes osteogenic differentiation of hBMSCs by targeting EZH2 and activating the Wnt/β-catenin pathway	Wang et al. (2016b)
	miR-101 is downregulated in eRMS, inversely correlated with EZH2, and its re-expression suppresses EZH2, reducing migration, clonogenicity, and tumorigenic potential of eRMS cells	Vella et al., (2015)
	miR-101 suppresses Ezh2, reducing invasion and migration of prostate cancer cells, with its expression further modulated by androgen signaling and HIF-1α/β induction	Cao et al. (2010)
	miR-101 downregulation drives EZH2 overexpression in GBM, and inhibiting EZH2 suppresses tumor growth, invasion, and angiogenesis both *in vitro* and *in vivo*	Smits et al. (2010)
	Re-expression of miR-101 suppresses proliferation, invasion, and self-renewal of aggressive endometrial cancer cells by directly targeting EZH2, MCL-1, and FOS	Konno et al. (2014)
	miR-101 is downregulated in bladder TCC, and its restoration suppresses proliferation by directly repressing EZH2, highlighting its role as a tumor suppressor	Friedman et al. (2009)
	miR-101 is downregulated in CRC, and its restoration suppresses EZH2-driven migration of colorectal cancer cells	Huang et al. (2021)
	Genomic loss of miR-101 in prostate cancer reduces its repression of EZH2, leading to EZH2 overexpression and epigenetic dysregulation that drives tumor progression	Varambally et al. (2008)
PTGS2/COX2	miR-101a directly repress COX-2 translation, with precursor overexpression reducing and antisense inhibition restoring COX-2 reporter and protein levels	Chakrabarty et al. (2007)
	miR-101a upregulation during mammary gland development suppresses COX-2, thereby inhibiting proliferation and modulating differentiation of mammary epithelial cells	Tanaka et al. (2009)
	miR-101 is downregulated in endometrial cancer, and its restoration suppresses angiogenesis and tumor growth partly through COX-2 regulation	Liu et al. (2018)
	Exogenous miR-101 directly targets COX-2, suppressing prostate cancer cell proliferation and reducing tumor growth *in vitro* and *in vivo*	Hao et al. (2011)
	miR-101 directly inhibits COX-2 translation, and its downregulation correlates with COX-2 overexpression in colorectal cancer cells and patient tissues	Strillacci et al. (2009)
	CdCl_2_ induces ER stress-driven COX-2/VEGF upregulation causing abnormal angiogenesis and cytotoxicity, which can be rescued by miR-101, siPTGS2, or COX-2 inhibition	Che et al. (2021)
	miR-101–3p suppresses breast cancer cell transmigration across the brain endothelium by downregulating COX-2/MMP1 signaling, thereby preserving junctional integrity and reducing brain metastasis potential	Harati et al. (2020)
	EPA upregulates miR-101 via the 15-LOX-1 pathway, leading to COX-2 suppression and inhibiting colon cancer progression	Cai et al. (2020)
ZEB1	miR-101a directly targets and suppresses ZEB1, thereby inhibiting epithelial-mesenchymal transition (EMT), reducing invasion and migration, and functioning as a tumor-suppressive regulator	Fan et al. (2019)
	ZEB1-AS1 promotes CRC proliferation and migration by sponging miR-101 to upregulate ZEB1, while miR-101 restoration or ZEB1-AS1 depletion suppresses these effects	Xiong et al. (2018)
	miR-101 directly suppresses ZEB1 expression, thereby inhibiting epithelial-to-mesenchymal transition (EMT), reducing cell migration, and limiting metastatic potential	Liang et al. (2018)
	M2-TAM-derived EVs deliver NEAT1, which sponges miR-101–3p to upregulate ZEB1/PD-L1, thereby promoting ovarian cancer growth and inducing CD8^+^ T Cell apoptosis	Yin and Wang (2023)
MCL-1/2	miR-101 suppresses Mcl-1, inhibits A549 lung cancer cell growth, and synergistically enhances sensitivity to etoposide by promoting apoptosis	Shahverdi et al. (2021)
	miR-101 suppresses MCL-1 by targeting its 3′-UTR, thereby promoting apoptosis and enhancing chemosensitivity	Wang et al. (2018b)
	Overexpression of miR-101 sensitizes papillary thyroid carcinoma cells to TRAIL-induced apoptosis by targeting c-Met and MCL-1 and inhibiting the PI3K/AKT pathway	Zhu and Li (2017)
CXCL6	Overexpression of miR-101–5p suppresses cervical cancer proliferation, migration, invasion, and tumor growth by directly targeting CXCL6	Shen et al. (2019)
	Overexpression of miR-101–5p suppresses NSCLC growth, invasion, and metastasis by directly targeting CXCL6	Chen et al. (2019a)
CXCL12	miR-101 is downregulated in PTC and suppresses proliferation, survival, migration, and invasion by directly targeting CXCL12 and inhibiting its downstream Akt/EMT signaling	Chen et al. (2019b)
MAT2A	hsa_circ_0007364 drives cervical cancer progression by sponging miR-101–5p, thereby relieving suppression of MAT2A and enhancing tumor cell proliferation, invasion, and growth	Chen et al. (2020a)
KPNA2	miR-101–3p is downregulated in cervical squamous cell carcinoma, and its overexpression suppresses tumor cell growth by directly targeting KPNA2	Wang et al. (2021)
mTOR	miR-101–3p enhances radiosensitivity of NSCLC by inhibiting the mTOR signaling pathway, reducing survival and promoting apoptosis in irradiated cells	Li et al. (2020a)
	lncRNA FAM201 A decreases radiosensitivity in ESCC by suppressing miR-101a, which in turn upregulates mTOR (and ATM) signaling	Chen et al. (2018)
ABCC1	Exosomal circ_PIP5K1A promotes NSCLC progression and cisplatin resistance by sponging miR-101a, which normally suppresses ABCC1 expression to inhibit tumor growth and enhance chemosensitivity	Shao et al. (2021)
CUL4B	miR-101a directly targets CUL4B, and its suppression by lncRNA SNHG12 enhances CUL4B expression, thereby promoting proliferation, migration, and invasion in non-small cell lung cancer	Xie and Liu (2021)
	circZFR promotes NSCLC progression by sponging miR-101a-3p, thereby relieving its repression of CUL4B and enhancing proliferation, migration, and invasion	Zhang et al. (2019)
​	miR-101a directly targets CUL4B, and its overexpression suppresses prostate cancer cell proliferation, migration, and invasion while promoting apoptosis by inhibiting the PI3K/AKT/mTOR pathway	Gu et al. (2021)
CDYL	SNHG6 promotes NSCLC growth and invasion by suppressing miR-101a-3p, thereby relieving its inhibition on CDYL.	Li et al. (2020b)
KRAS	miR-101a directly targets the KRAS 3′UTR, and its repression by circ-MEMO1 leads to KRAS upregulation, thereby promoting proliferation, glycolysis, and tumor growth in NSCLC.	Ding et al. (2020)
TRIM44	miR-101–3p directly targets TRIM44, suppressing EMT and thereby reducing proliferation, migration, and invasion of glioblastoma cells	Li et al. (2019)
RAP1A/B	miR-101a suppresses Rap1A expression in prostate cancer, an effect antagonized by lncRNA CRNDE, thereby restraining proliferation, migration, and invasion	Chen et al. (2020b)
	miR-101a suppresses colorectal cancer progression by directly targeting Rap1b, forming a negative feedback loop that inhibits proliferation, migration, and invasion	Zhou et al. (2020)
HDAC9	miR-101a-3p suppresses retinoblastoma cell proliferation by directly targeting HDAC9, and restoring HDAC9 reverses this anti-proliferative effect	Jin et al. (2018)
Girdin	miR-101a acts as a tumor suppressor in HCC by directly targeting Girdin, thereby inhibiting cell proliferation, migration, and invasion	Cao et al. (2016)
VEGF-C	miR-101a suppresses HCC cell migration and invasion by directly targeting VEGF-C, highlighting its role in restraining cytoskeletal remodeling and girding during metastasis	Liu et al. (2016b)
	miR-101a suppresses VEGF-C, and its inhibition by MALAT1 promotes cisplatin resistance in bladder cancer cells	Liu et al. (2019)
	In cholangiocarcinoma, miR-101a is underexpressed, correlating with VEGF overexpression, suggesting its loss may promote tumor progression and reduced survival	Calastri et al. (2022)
	miR-101a-3p suppresses VEGFA in cancer-associated fibroblasts, thereby reducing CAF-driven EMT, invasion, and metastasis in lung cancer	Guo et al. (2021)
	miR-101a suppresses cholangiocarcinoma growth by directly targeting VEGF and indirectly repressing its transcription via COX-2 inhibition, thereby blocking angiogenesis	Zhang et al. (2013)
ZO-1	miR-101a-3p, upregulated by ethanolamine accumulation in obesity, destabilizes ZO-1 mRNA, thereby weakening intestinal barriers and promoting gut permeability, inflammation, and metabolic dysfunction	Mishra et al. (2023)
CLDN1	miR-101a suppresses CLDN1 expression to inhibit papillary thyroid carcinoma cell migration and invasion, while XIST promotes metastasis by sponging miR-101a and restoring CLDN1	Du et al. (2021)
ZNF217	miR-101 suppresses ZNF217 in hepatocellular carcinoma, thereby restoring CDH1 expression and inhibiting proliferation, EMT, and invasion	Si et al. (2019)
c-Met pathway	miR-101a sensitizes papillary thyroid carcinoma cells to TRAIL by targeting c-Met (and MCL-1), thereby inhibiting PI3K/AKT signaling and reducing apoptosis resistance	Zhu and Li (2017)
c-Met pathway MAPK/ERK pathway	miR-101a suppresses tumor growth and progression by downregulating pro-angiogenic signaling, such as VEGF-related pathways, through targeting upstream regulators like the HGF/c-MET axis	Liu et al. (2020)
	miR-101a is downregulated in liver cancer, and its overexpression suppresses proliferation by targeting EZH2 and inhibiting the MAPK/ERK signaling pathway	Meng et al. (2020)
	miR-101a, enriched in the brain, promotes differentiation of bone marrow cells into microglia-like cells by enhancing inflammatory responsiveness and downregulating MAPK phosphatase-1	Saika et al. (2017)
Lin28 B	In NSCLC, IL-1β suppresses miR-101, leading to Lin28 B upregulation and enhanced proliferation and migration, an effect reversible by COX-2 inhibition	Wang et al. (2014a)
ANXA2	miR-101a suppresses ANXA2, thereby inhibiting ERK signaling, reducing LCSC proliferation and metastasis, and forming a regulatory miR-101/ANXA2/EGR1 loop in liver cancer	Ma et al. (2021)
SRF	miR-101a suppresses gastric adenocarcinoma cell proliferation and invasion by directly targeting SRF, thereby downregulating HOTAIR transcription	Ma et al. (2021)
PIM 1	miR-101a suppresses PIM1 expression in gastric cancer cells, thereby inhibiting proliferation and invasion while promoting apoptosis	Wu et al. (2019)
FZD4	miR-101a is downregulated in bladder cancer, and its restoration suppresses migration and invasion by directly targeting FZD4	Chen et al. (2019c)
STMN1	miR-101a suppresses pancreatic cancer cell proliferation and invasion by directly targeting and downregulating STMN1	Zhu et al. (2018)
HIPK3	miR-101a-3p acts as an oncomiR in colorectal cancer by targeting HIPK3, thereby enhancing cell growth, migration, glycolysis, and reducing chemosensitivity	Tao et al. (2021)
Notch1	miR-101a suppresses NOTCH1 signaling, while CircAPLP2 promotes colorectal cancer proliferation and metastasis by sponging miR-101a and thereby reactivating the Notch pathway	Wu et al. (2020)
CREB1	miR-101a is downregulated in colon cancer, and its overexpression suppresses proliferation and migration by directly targeting CREB1	Yang et al. (2019)
BICC1	miR-101a is downregulated in oral cancer, and its restoration suppresses BICC1, thereby reducing cell viability and promoting apoptosis	Wang et al. (2020)
Jak2	miR-101a suppresses proliferation and induces apoptosis in breast cancer cells by directly targeting Jak2	Wang et al. (2014b)

Abbreviation: ABCC1, ATP-binding cassette subfamily C member 1; AKT, protein kinase B; ANXA2, annexin A2; ATM, ataxia telangiectasia mutated; BICC1, bicaudal C homolog 1; CAF, cancer-associated fibroblast; CDH1 (E-cadherin), epithelial cadherin; CDYL, chromodomain Y-like protein; CLDN1, claudin-1; COX-2 (PTGS2), cyclooxygenase-2 (prostaglandin-endoperoxide synthase 2); CREB1, cAMP, response element-binding protein 1; CRC, colorectal cancer; CRNDE, colorectal neoplasia differentially expressed; CUL4B, cullin 4B; CXCL6, C-X-C motif chemokine ligand 6; CXCL12, C-X-C motif chemokine ligand 12; DNMT3A, DNA, methyltransferase three alpha; EMT, epithelial-mesenchymal transition; EPA, eicosapentaenoic acid; ERK, extracellular signal-regulated kinase; eRMS, embryonal rhabdomyosarcoma; EVs, extracellular vesicles; EZH2, enhancer of zeste homolog 2; FOS, FBJ, murine osteosarcoma viral oncogene homolog; FZD4, frizzled class receptor 4; GBM, glioblastoma multiforme; Girdin (CCDC88 A), coiled-coil domain-containing protein 88A; HDAC9, histone deacetylase 9; HGF, hepatocyte growth factor; HIF-1α/β, hypoxia-inducible factor-1, alpha/beta; HIPK3, homeodomain-interacting protein kinase 3; HOTAIR, HOX, transcript antisense RNA; IL-1β, interleukin-1, beta; JAK2, Janus kinase 2; KPNA2, karyopherin subunit alpha 2; KRAS, kirsten rat sarcoma viral oncogene homolog; Lin28B, Lin-28, homolog B; MALAT1, metastasis-associated lung adenocarcinoma transcript 1; MAPK, mitogen-activated protein kinase; MAT2A, methionine adenosyl transferase 2A; MCL-1, myeloid cell leukemia sequence 1; MMP1, matrix metalloproteinase-1; mTOR, mechanistic target of rapamycin; NEAT1, nuclear paraspeckle assembly transcript 1; NSCLC, non-small cell lung cancer; PD-L1, programmed death-ligand 1; PI3K, phosphoinositide 3-kinase; PIM1, proviral integration site for Moloney murine leukemia virus 1; RAP1A/B, Ras-related protein 1A/1B; SNHG12, small nucleolar RNA, host gene 12; SRF, serum response factor; STMN1, stathmin 1; TAM, tumor-associated macrophage; TCC, transitional cell carcinoma; TRAIL, tumor necrosis factor-related apoptosis-inducing ligand; TRIM44, tripartite motif-containing protein 44; VEGF, vascular endothelial growth factor; VEGF-C, vascular endothelial growth factor C; Wnt/β-catenin, Wingless/Integrated beta-catenin signaling pathway; XIST, X-inactive specific transcript; ZEB1, zinc finger E-box binding homeobox 1; ZEB1-AS1, ZEB1 antisense RNA, 1; ZNF217, zinc finger protein 217; ZO-1 (TJP1), zonula occludens-1 (tight junction protein 1).

### Molecular targets of miR-101a and their roles in colorectal tumorigenesis

**FIGURE 2:**
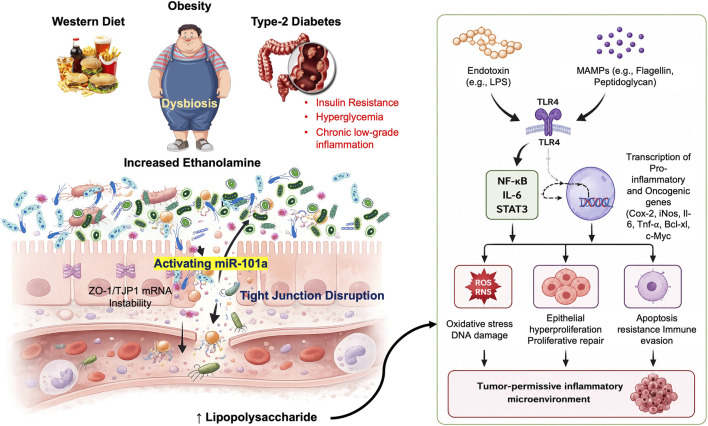
Proposed molecular mechanism linking ethanolamine-induced miR-101a dysregulation to obesity-associated colorectal carcinogenesis. Schematic representation illustrating the proposed pathway connecting Western diet-associated metabolic dysregulation to colorectal tumorigenesis through the ethanolamine–miR-101a axis. Western diet, obesity, and T2D promote gut microbial dysbiosis, insulin resistance, hyperglycemia, and chronic low-grade inflammation, resulting in increased luminal ethanolamine and enrichment of pro-inflammatory microbial products. Elevated ethanolamine induces miR-101a expression in colonic epithelial cells, leading to destabilization of ZO-1/TJP1 mRNA and disruption of epithelial tight-junction integrity. The resulting epithelial barrier dysfunction increases intestinal permeability, endotoxemia, and the translocation of microbial products, including lipopolysaccharide (LPS) and other microbe-associated molecular patterns (MAMPs). These microbial signals activate TLR4-mediated inflammatory pathways involving NF-κB, IL-6, and STAT3 signaling, which promote transcription of pro-inflammatory and oncogenic mediators, including COX-2, iNOS, TNF-α, Bcl-xL, and c-Myc. Sustained inflammatory activation further induces oxidative stress, DNA damage, epithelial hyperproliferation, proliferative repair, apoptosis resistance, and immune evasion, collectively establishing a tumor-permissive inflammatory microenvironment that may facilitate adenoma initiation and progression toward obesity-associated colorectal cancer. miR-101a is positioned as a central microbiota-responsive mediator linking ethanolamine metabolism, epithelial barrier dysfunction, chronic inflammation, and tumor-promoting signaling pathways in obesity/T2D-associated colorectal carcinogenesis.

In the colon and mammary epithelium, the miR-101 family regulates ZO-1 mRNA stability, COX-2 signaling, and cytoskeletal programs, thereby indirectly linking it to barrier and mucosal layer integrity and epithelial restitution (Chandramouli et al., 2012; Tâlvan et al., 2024; Strillacci et al., 2009; Schaefer et al., 2015). In parallel, its developmental trajectory and neuronal expression support roles in synaptic maturation and activity-dependent plasticity. Together, genomic organization, sequence features, and variant landscapes position the miR-101 family as a precision node through which diet-microbe-host signals (e.g., ethanolamine-driven transcriptional shifts) can recalibrate target accessibility and pathway flux, with implications for context-dependent phenotypes in health and disease (Figure 2). Chronic inflammation further recruits immunosuppressive cells, such as regulatory T-cells and myeloid-derived suppressor cells, which dampen anti-tumor immunity and create a permissive environment for malignant clones (Greten and Grivennikov, 2019; Umansky et al., 2016). This shift from immune surveillance to immune evasion marks a critical step in tumor progression, allowing adenomas to potentially progress toward invasive carcinomas.

The role of the miR-101 family in CRC is highly microenvironment-specific, which explains its paradoxical classification as both a tumor suppressor and a context-dependent pro-inflammatory regulator. In many non-obese contexts, loss of miR-101a contributes to tumorigenesis by relieving repression of oncogenes, including MCL-1, EZH2, Rap1b, and DNMT3A (Konno et al., 2014; Varambally et al., 2008; Yan et al., 2014; Zhou et al., 2020). In contrast, in the obese and dysbiotic colon, over-expression of miR-101a acts through an entirely different mechanism, weakening epithelial barrier defenses, fueling chronic inflammation (Mishra et al., 2023), thereby indirectly contributing to carcinogenesis-supportive microenvironmental conditions. This duality highlights the importance of considering metabolic, microbial, and inflammatory signals when assessing miRNA function in cancer biology. Barrier dysfunction increases stem cell exposure to microbial ligands and mutagens, inflammation sustains proliferative and survival signaling, and microenvironmental remodeling accelerates progression. In this way, miR-101a is integrated into the canonical CRC model as a non-genetic, context-dependent regulator that may increase the likelihood of malignant transformation in metabolically stressed environments.

Emerging clinical evidence indicates that circulating and tissue levels of human miR-101-1/2 are altered in CRC and may have diagnostic or prognostic relevance. Several studies have reported that miR-101-1 expression is frequently reduced in CRC tissues and patient serum, consistent with its widely recognized tumor-suppressive function. For example, Zhou et al. (2020) demonstrated that miR-101-1 expression is significantly decreased in CRC tissues compared with adjacent normal mucosa, and that restoration of miR-101 suppresses tumor cell proliferation and invasion by directly targeting Rap1b, a regulator of cell migration and oncogenic signaling (Zhou et al., 2020). Similarly, Yang et al., 2019 showed that reduced miR-101-1 expression in colon cancer tissues correlates with enhanced tumor growth and invasive potential by regulating CREB1-dependent transcriptional pathways, highlighting its functional importance in colorectal tumor progression (Yang et al., 2019). More recently, Tâlvan et al., 2025 performed clinical profiling of miRNA expression in CRC patients and reported that altered levels of miR-101-1/2 are associated with tumor grade, invasiveness, and anatomical localization, suggesting that miR-101 expression patterns may reflect disease severity and biological heterogeneity in colorectal cancer (Tâlvan et al., 2025). Collectively, these studies support the concept that reduced circulating or tissue levels of the miR-101 family are associated with aggressive tumor phenotypes and poorer clinical outcomes, suggesting that this miRNA may serve as a potential diagnostic or prognostic biomarker in CRC. Importantly, these clinical observations also highlight the context-dependent nature of miR-101-1/2 biology, suggesting that while loss of miR-101-1/2 is commonly linked to tumor progression, altered regulation of miR-101-1/2 in metabolically dysregulated environments may contribute to distinct mechanisms of disease development.

## Therapeutic and biomarker potential of the miR-101 family

Beyond its mechanistic involvement in epithelial barrier dysfunction and inflammatory remodeling, the miR-101 family is increasingly emerging as a clinically actionable biomarker and therapeutically targetable regulator in cancer biology. Human miR-101-1/2 expression profiles are increasingly recognized as translationally relevant biomarkers because miRNAs remain highly stable in serum, plasma, stool, and extracellular vesicles, supporting their utility in minimally invasive CRC detection and disease monitoring (Pastena et al., 2024; Kowalczuk et al., 2023). Importantly, recent clinical studies have demonstrated that altered expression of human miR-1011/2 correlates with CRC severity, invasiveness, and tumor heterogeneity, suggesting potential diagnostic and prognostic relevance for CRC patient stratification (Tâlvan et al., 2024; Tâlvan et al., 2025).

Beyond biomarker applications, the miR-101 family regulates multiple therapeutically relevant pathways associated with epithelial integrity, inflammatory signaling, epigenetic remodeling, and tumor progression, including EZH2, COX-2/PTGS2, DNMT3A/B, MCL-1, and STMN1 (Cao et al., 2011; Liu et al., 2022; Konno et al., 2014; Wang C. Z. et al., 2018; Huang et al., 2021; Zhu et al., 2018). These findings position the miR-101 family as a mechanistically integrated regulator of tumor-permissive signaling rather than a single-pathway effector. In murine systems, miR-101a/b-mediated epithelial barrier disruption and inflammatory activation are particularly relevant under obesity-associated and dysbiotic conditions, where microbiota-derived ethanolamine induces miR-101a overexpression and destabilizes ZO-1/TJP1 (Mishra et al., 2023). Collectively, these observations suggest that selective modulation of the ethanolamine-miR-101a axis may represent a precision therapeutic strategy targeting upstream inflammatory and barrier dysfunction pathways involved in obesity-associated CRC.

Recent advances in RNA therapeutics further support the translational potential of miR-101 family-directed interventions. Antisense oligonucleotide-locked nucleic acid (LNA)- based inhibitors, miRNA mimetics, exosome-mediated delivery systems, and lipid nanoparticle RNA platforms are increasingly being explored in cancer therapeutics (Singh et al., 2026; Yan et al., 2025). Given the context-dependent functions of the miR-101 family, future therapeutic approaches will likely require tissue-specific and metabolic-state-specific modulation rather than universal inhibition or overexpression strategies. In addition, microbiome-directed interventions targeting ethanolamine-utilizing (eut) operon metabolism, engineered probiotics, and postbiotic therapies may provide complementary approaches for indirectly regulating miR-101 family-associated inflammatory and epithelial barrier pathways. Potential approaches include microbiome-based interventions, such as ethanolamine-utilizing probiotics (e.g., *Lactobacillus rhamnosus* HL-200), modulation of microbial metabolites, and selective targeting of miR-101a using inhibitors or mimetics (Mishra et al., 2023).

Collectively, these emerging concepts position the miR-101 family as a promising component of next-generation precision oncology frameworks integrating microbiome biology, epithelial barrier function, inflammatory signaling, and RNA therapeutics. Future translational studies incorporating longitudinal human cohorts, single-cell and spatial transcriptomics, organoid systems, and microbiome-integrated multi-omics analyses will be essential to determine whether miR-101-directed interventions can meaningfully improve risk prediction, therapeutic responsiveness, or disease outcomes in obesity-associated CRC.

## Limitation and future direction

Several limitations should be considered when interpreting the proposed ethanolamine/murine miR-101a in obesity/T2D-associated CRC, while simultaneously highlighting critical opportunities for future investigation and therapeutic innovation. Current evidence supports a role for ethanolamine-induced upregulation of miR-101a in intestinal barrier dysfunction (ZO-1) and metabolic inflammation, particularly through disruption of epithelial tight-junction integrity and activation of inflammatory signaling pathways. However, direct experimental evidence demonstrating that this pathway causally influences conditions associated with colorectal tumor initiation, adenoma burden, dysplasia progression, or invasive CRC outcomes in obesity-specific settings remains limited. Therefore, the proposed axis should be interpreted as a mechanistically plausible and hypothesis-generating framework, rather than a definitively established contributor to colorectal carcinogenesis. A central challenge lies in the paradoxical and context-dependent role of miR-101a. While a substantial body of literature describes miR-101 family as a tumor suppressor in multiple cancer settings, the tumor-promoting interpretation proposed here appears to be restricted to metabolically dysregulated and microbiota-dependent environments. The mechanisms underlying this functional switch remain incompletely understood and likely depend on epithelial cell context, microbial composition, metabolite availability, and disease stage. In addition, current clinical observations are heterogeneous and often derived from relatively small or insufficiently stratified cohorts, underscoring the need for larger, well-characterized human studies. Importantly, direct human evidence linking intestinal or circulating ethanolamine levels with miR-101 family expression, epithelial barrier dysfunction, inflammatory biomarkers, and CRC progression in obesity-associated cohorts remains lacking and should represent a major priority for future translational and multi-omics investigations. Although several microbiota-derived metabolites, including SCFAs and bile acids, are known to influence epithelial homeostasis, inflammation, and colorectal carcinogenesis, direct mechanistic evidence linking these metabolites to miR-101 family regulation remains limited. Currently, the strongest available evidence supports microbiota-derived ethanolamine as an inducer of murine miR-101a under obesity-associated dysbiotic conditions, while the potential contribution of other microbial metabolites to miR-101 family regulation requires further investigation. Emerging evidence also suggests that microbial quorum-sensing molecules may influence epithelial signaling, inflammation, and host transcriptomic regulation; however, their interactions with miR-101 family signaling in obesity-associated CRC remain largely unexplored and warrant further investigation.

These limitations define important opportunities for future research and therapeutic development. The dual role of miR-101a suggests that simple inhibition or overexpression may not be sufficient. Instead, condition-specific and precision-based therapeutic strategies are required, and rigorous experimental validation of the ethanolamine-miR-101a axis in obesity-associated CRC models is essential. This includes the use of genetically engineered mouse models (e.g., miR-101a gain- and loss-of-function in APC^Min/+^ backgrounds), gnotobiotic systems, fecal microbiota transplantation approaches, and patient-derived organoid platforms to establish causal relationships and tissue-specific effects. Longitudinal and stratified clinical studies will also be required to determine whether miR-101a-associated barrier dysfunction correlates with disease risk, progression, or therapeutic response in humans. From a translational perspective, the context-dependent nature of miR-101a suggests that precision-based and environment-specific interventions will be necessary. Potential strategies include modulation of microbial metabolism (e.g., targeting ethanolamine-utilizing pathways), microbiome-directed interventions, and selective targeting of miR-101a using inhibitors or mimetics. However, these approaches remain conceptual at this stage and require systematic preclinical validation to establish efficacy, safety, and context specificity.

Moreover, integrating microbiome modulation with miRNA targeting represents a novel therapeutic direction with strong translational potential. This approach may enable precision medicine guided by biomarker signatures, in which miR-101a/microbiome signatures define treatment response. It also provides a basis for the development of innovative and potentially therapeutic agents. Overall, future studies should define miR-101a as a context-dependent regulator of barrier function and inflammation and critically evaluate its potential as both a biomarker and a therapeutic target in obesity-associated CRC.

## Conclusion

Taken together, current evidence supports context-dependent roles of the miR-101 family, particularly murine miR-101a as a regulator of epithelial barrier dysfunction and inflammation in obesity-associated CRC. Rather than serving as a direct mediator of tumor initiation or progression, miR-101a appears to contribute to the establishment of a tumor-permissive microenvironment by disrupting epithelial integrity, enhancing intestinal permeability, and sustaining inflammatory signaling. By linking dietary metabolites, such as ethanolamine, to microbial dysbiosis and host epithelial regulation, miR-101a exemplifies the intricate interactions that increase the risk of colorectal adenomas beyond genetic mutations alone. Importantly, while this ethanolamine-miR-101a axis provides a biologically plausible and mechanistically coherent framework connecting metabolic dysregulation to colorectal carcinogenesis, direct experimental evidence demonstrating its causal role in adenoma formation, dysplasia progression, or invasive CRC in obesity-specific models remains limited and requires further validation in well-defined preclinical systems and clinical cohorts. This perspective underscores the need for context-specific investigation in miRNA biology, as the same molecule may function as a tumor suppressor or promoter based on metabolic, microbial, and tissue-specific conditions. In obesity-associated CRC, miR-101a emerges as a context-dependent molecular bridge linking diet, microbiota, and epithelial dysfunction, as well as inflammatory microenvironmental remodeling, offering both mechanistic insight and translational relevance. The ethanolamine-miR-101a axis should thus be viewed as one component of a broader, multifactorial network of obesity-associated carcinogenic pathways. Additional contributors include the genotoxic effects of dietary carcinogens, chronic systemic inflammation driven by adipokines and cytokines, and microbial production of pro-carcinogenic metabolites. Together, these factors interact to shape a complex tumor-permissive landscape. Finally, the context-dependent duality of miR-101a highlights its potential as both a biomarker and a therapeutic target. Future studies leveraging obesity-associated CRC models, longitudinal human cohorts, and integrative multi-omics approaches will be essential to determine whether modulation of this axis can influence disease risk or progression, thereby informing the development of precision microbiome-miRNA-targeted interventions.

## Glossary

ABCC1: ATP-Binding Cassette Subfamily C Member 1
AKT: Protein Kinase B
ANXA2: Annexin A2
AP-1: Activator Protein 1
ATM: Ataxia Telangiectasia Mutated
BCL-2: B-Cell Lymphoma 2
BICC1: Bicaudal C Homolog 1
CAF: Cancer-Associated Fibroblast
CDH1 (E-cadherin): Cadherin 1
CDK8: Cyclin-Dependent Kinase 8
CDYL: Chromodomain Y-Like Protein
CLDN1: Claudin-1
COX-2 (PTGS2): Cyclooxygenase-2 (Prostaglandin-Endoperoxide Synthase 2)
CREB1: cAMP Response Element-Binding Protein 1
CRC: Colorectal Cancer
CRNDE: Colorectal Neoplasia Differentially Expressed
CUL4B: Cullin 4B
CXCL6: C-X-C Motif Chemokine Ligand 6
CXCL12: C-X-C Motif Chemokine Ligand 12
DDR: DNA Damage Response
DGCR8: DiGeorge Syndrome Critical Region 8
DNA-PKcs: DNA-Dependent Protein Kinase Catalytic Subunit
DNMT: DNA Methyltransferase
DNMT3A: DNA Methyltransferase 3 Alpha
DNMT3B: DNA Methyltransferase 3 Beta
DOT1L: Disruptor of Telomeric Silencing 1-Like
EMT: Epithelial-Mesenchymal Transition
EPA: Eicosapentaenoic Acid
ERK: Extracellular Signal-Regulated Kinase
eRMS: Embryonal Rhabdomyosarcoma
EVs: Extracellular Vesicles
EZH2: Enhancer of Zeste Homolog 2
FOS: FBJ Murine Osteosarcoma Viral Oncogene Homolog
FZD4: Frizzled Class Receptor 4
GBM: Glioblastoma Multiforme
Girdin (CCDC88A): Coiled-Coil Domain-Containing Protein 88A
HDAC9: Histone Deacetylase 9
HGF: Hepatocyte Growth Factor
HIF-1α/β: Hypoxia-Inducible Factor 1 Alpha/Beta
HIPK3: Homeodomain-Interacting Protein Kinase 3
HOTAIR: HOX Transcript Antisense RNA
IL: Interleukin
IL-1β: Interleukin-1 Beta
IL-6: Interleukin-6
JAK2: Janus Kinase 2
KPNA2: Karyopherin Subunit Alpha 2
KRAS: Kirsten Rat Sarcoma Viral Homolog
LPS: Lipopolysaccharide
Lin28B: Lin-28 Homolog B
lncRNA: Long Non-Coding RNA
MALAT1: Metastasis-Associated Lung Adenocarcinoma Transcript 1
MAPK: Mitogen-Activated Protein Kinase
MAT2A: Methionine Adenosyltransferase 2A
MAMPs: Microbe-Associated Molecular Patterns
MCL-1: Myeloid Cell Leukemia Sequence 1
MMP1: Matrix Metalloproteinase 1
mRNA: Messenger RNA
miRNA: MicroRNA
MUC2: Mucin 2
mTOR: Mechanistic Target of Rapamycin
NEAT1: Nuclear Paraspeckle Assembly Transcript 1
NF-κB: Nuclear Factor Kappa-Light-Chain-Enhancer of Activated B Cells
NSCLC: Non-Small Cell Lung Cancer
PD-L1: Programmed Death Ligand 1
PI3K: Phosphoinositide 3-Kinase
PIM1: Proviral Integration Site for Moloney Murine Leukemia Virus 1
PRC2: Polycomb Repressive Complex 2
PRRs: Pattern Recognition Receptors
PTC: Papillary Thyroid Carcinoma
RAP1A/B: Ras-Related Protein 1A/1B
Rcl1: RNA Terminal Phosphate Cyclase-Like 1
RISC: RNA-Induced Silencing Complex
ROS: Reactive Oxygen Species
RNS: Reactive Nitrogen Species
SNHG12: Small Nucleolar RNA Host Gene 12
SOX9: SRY-Box Transcription Factor 9
SRF: Serum Response Factor
STAT3: Signal Transducer and Activator of Transcription 3
STMN1: Stathmin 1
T2D: Type 2 Diabetes
TAM: Tumor-Associated Macrophage
TCC: Transitional Cell Carcinoma
TLR: Toll-Like Receptor
TNF-α: Tumor Necrosis Factor Alpha
TRAIL: Tumor Necrosis Factor-Related Apoptosis-Inducing Ligand
TRIM44: Tripartite Motif-Containing Protein 44
VEGF: Vascular Endothelial Growth Factor
VEGF-C: Vascular Endothelial Growth Factor C
Wnt/β-catenin: Wingless/Integrated Beta-Catenin Signaling Pathway
XIST: X-Inactive Specific Transcript
ZEB1: Zinc Finger E-Box Binding Homeobox 1
ZEB1-AS1: ZEB1 Antisense RNA 1
ZNF217: Zinc Finger Protein 217
ZO-1 (TJP1): Zonula Occludens-1 (Tight Junction Protein 1)
